# Effects of Unilateral Nordic Hamstring Training with a Sloped Platform on Biceps Femoris Fascicle Length in Athletes with a History of Hamstring Strain Injury

**DOI:** 10.3390/muscles5020022

**Published:** 2026-03-25

**Authors:** Toshiaki Soga, Taspol Keerasomboon, Parunchaya Jamkrajang, Norikazu Hirose

**Affiliations:** 1 Graduate School of Engineering and Science, Shibaura Institute of Technology, Saitama 337-8570, Japan; 2 Japan Society for the Promotion of Science, Tokyo 102-0083, Japan; 3 College of Sports Science and Technology, Mahidol University, Nakhon Pathom 73170, Thailand; taspol.kee@mahidol.edu (T.K.); parunchaya.jam@mahidol.edu (P.J.); 4 Faculty of Sport Sciences, Waseda University, Tokyo 202-0021, Japan; toitsu_hirose@waseda.jp

**Keywords:** injury prevention, recurrence prevention, hamstring muscles, muscle thickness, eccentric strength

## Abstract

Research has yet to investigate the effects of unilateral Nordic hamstring training with a sloped platform (UNHT) on the fascicle length of the biceps femoris long head (BFlh FL) in previously injured legs. This study aimed to address this gap. A longitudinal pre–post design without a control group was used to examine the effects of 6 weeks of UNHT in eight young male athletes with a history of hamstring strain injury (HSI). Pre- and post-training measurements of middle and distal BFlh FL in the injured and uninjured legs were obtained using ultrasonography. At baseline, middle BFlh FL was significantly shorter in the injured leg than in the uninjured leg (86.0 ± 9.0 mm vs. 99.9 ± 7.6 mm, *p* < 0.001). Following UNHT, middle BFlh FL significantly increased in both the injured (105.8 ± 9.4 mm, *p* = 0.001) and uninjured legs (108.0 ± 10.3 mm, *p* = 0.596). Distal BFlh FL significantly increased in the injured leg (72.4 ± 7.1 mm to 85.7 ± 8.0 mm, *p* = 0.012), whereas the uninjured leg showed a smaller, non-significant change (72.8 ± 5.5 mm to 79.1 ± 7.5 mm, *p* = 0.069). These findings demonstrate that UNHT increased BFlh FL in previously injured legs. Because shorter BFlh FL and reduced eccentric strength are established risk factors for HSI, these adaptations may have implications for risk reduction; however, recurrence prevention was not assessed in this study.

## 1. Introduction

Hamstring strain injuries (HSIs), commonly caused by sprinting and stretching activities, are frequent in football (soccer) [1,2]. Previous research has reported that the incidence rate of HSIs has doubled, from 12% to 24% of all injuries, between 2001 and 2022 in male professional soccer players [3]. A substantial concern with HSIs is their recovery timeline. Severe cases require approximately 1.5–3.5 months before the athletes can return to play [4], and over two-thirds of athletes experience recurrence within 2 months of resuming activity [3]. Therefore, preventing not only initial HSIs but also their recurrence is important to maintain athletic performance.

In soccer, the highest percentage of HSIs occurs in the biceps femoris long head (BFlh), with sprint-related HSIs accounting for 88–100% of cases and stretch-related HSIs comprising 60–70% [1,2]. Following an HSI, the fascicle length of the BFlh (BFlh FL) is shorter in the previously injured leg than in the uninjured leg [5,6]. Because reduced BFlh FL is a strong risk factor for HSI [7], this shortening may increase the likelihood of recurrence. Consequently, training interventions that preferentially load the BFlh and thereby stimulate fascicle lengthening are essential for preventing HSI recurrence.

The Nordic hamstring exercise (NHE) is performed to improve eccentric knee flexion strength [8]. This exercise involves maintaining a straight posture from the knees to the head while leaning the upper body forward to a level that the hamstring muscles can tolerate. The knee flexion angle at which knee flexor strength cannot resist the external knee flexion moment that occurs when the trunk leans forward is referred to as the break-point angle (BPA). The BPA is approximately 50° even in trained soccer players [9], indicating that hamstring activation may be limited at shallow knee flexion angles in many athletes during standard bilateral NHE [10]. Given that the BFlh is preferentially recruited at shallow knee flexion angles [11], standard bilateral NHE may not maximize BFlh recruitment [11].

Recent studies have explored the NHE using a sloped platform [10,11,12]. A sloped platform enables movement into shallow knee flexion positions, confirming high hamstring activity with preferential recruitment of the BFlh compared with the semitendinosus muscle (ST) [10]. Furthermore, unilateral NHE performed with a sloped platform has been shown to further increase BFlh recruitment compared with bilateral NHE with the same platform [12]. This combination—shallow knee angles and unilateral loading—may be particularly effective for inducing architectural adaptations. Specifically, eccentric loading at long muscle lengths is known to promote fascicle lengthening, and preferential recruitment of the BFlh at shallow angles increases the likelihood that the mechanical stimulus is directed toward this muscle, thereby strengthening the rationale for expecting BFlh FL adaptation.

Previously injured legs may respond more favorably to BFlh FL training effects than uninjured legs [5]. However, the impact of UNHT on BFlh FL in individuals with a history of HSI has not been investigated. Therefore, this study aimed to investigate the effects of UNHT with a sloped platform on the BFlh FL in previously injured legs. In particular, this study investigated how UNHT influences eccentric knee flexion strength and hamstring muscle hypertrophy.

The rationale for expecting architectural adaptation is based on the mechanical characteristics of UNHT. Performing the exercise on a sloped platform allows the athlete to reach shallow knee flexion angles, where the BFlh is preferentially recruited. This preferential activation increases the likelihood that eccentric loading is applied specifically to the BFlh at long muscle lengths—a condition known to promote fascicle lengthening through the addition of sarcomeres in series.

Furthermore, because strain distribution within the BFlh is non-uniform during eccentric loading, with distal regions often experiencing greater mechanical strain than middle regions, we considered the possibility of region-specific architectural adaptations. This provided the a priori rationale for examining middle and distal fascicle regions separately.

Based on these mechanisms, we hypothesized that UNHT would induce greater BFlh FL lengthening in previously injured legs than in uninjured legs.

## 2. Results

### 2.1. Participant Characteristics

Eight young male athletes (age 20.5 ± 1.6 years, height 177.5 ± 8.3 cm, and body mass 70.5 ± 10.6 kg) participated in this study. The sample consisted of athletes from taekwondo (n = 3), sepak takraw (n = 2), soccer (n = 2), and basketball (n = 1). All participants were actively engaged in competitive training, typically completing 4–6 structured training sessions per week, indicating a well-trained athletic population. We obtained information on the rate of sprint-related HSI (25.0%), rate of stretch-related HSI (75.0%), time since the last injury (13.3 ± 8.2 months; range: 1–24 months), and number of previous injuries (1.6 ± 0.7 times; range: 1–3 times) based on self-reports. The injured side was determined based solely on athletes’ self-reported history of clinically diagnosed HSIs; no medical or team records were available for verification. Participants completed 99.0% of the prescribed sessions (95 of 96 sessions).

### 2.2. Fascicle Length of the Biceps Femoris Long Head

**Figure 1:**
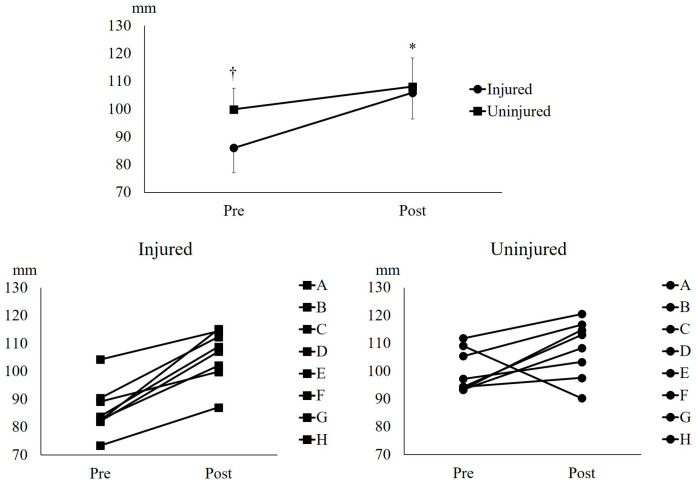
**Middle fascicle length of the biceps femoris long head.** † indicates *p* < 0.01 between the injured and uninjured legs. * indicates *p* < 0.01 between Pre and Post in the injured legs.

In the middle BFlh FL, results from the two-way analysis of variance (ANOVA) showed significant effects for interaction (*p* = 0.029, partial η^2^ = 0.519), group (*p* = 0.002, partial η^2^ = 0.753), and time (*p* = 0.019. partial η^2^ = 0.569). In the pre-training measurement, the middle BFlh FL of the uninjured leg was significantly longer than that of the injured leg (*p* = 0.000, d = 1.66) (Figure 1). The UNHT significantly increased the middle BFlh FL of the injured (*p* = 0.001, d = 2.16), whereas no significant change was observed in the uninjured leg (*p* = 0.596, d = 0.90) (Figure 1). The between-leg difference in change (injured − uninjured) for the middle BFlh fascicle length was 11.4 mm (95% CI: 1.4 to 21.3 mm), indicating greater architectural adaptation in the injured leg.

**Figure 2:**
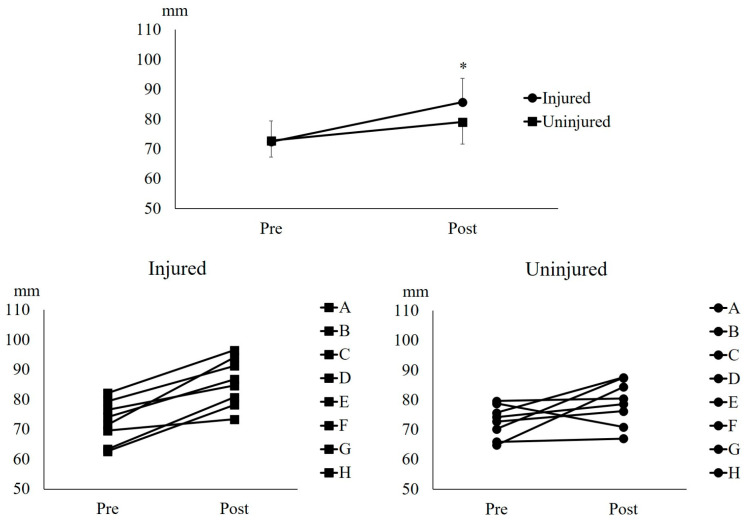
**Distal fascicle length of the biceps femoris long head.** * indicates *p* < 0.05 between Pre and Post in the injured legs.

The Wilcoxon signed-rank test showed that UNHT significantly increased the distal BFlh FL of the injured leg (*p* = 0.012, r = 0.89), but no significant increase was observed in the uninjured leg (*p* = 0.069, r = 0.64) (Figure 2).

### 2.3. Eccentric Knee Flexion Strength

**Figure 3:**
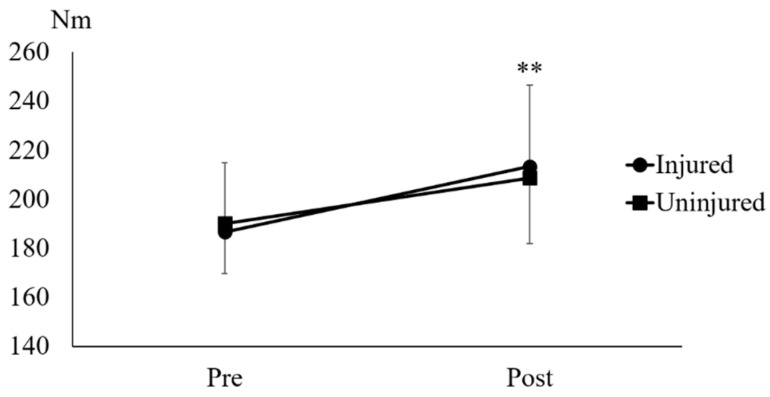
**Eccentric knee flexion strength.** ** indicates *p* < 0.01 between Pre and Post in the injured and uninjured legs.

The paired *t*-test showed that UNHT significantly increased the eccentric knee flexion strength in the injured (*p* = 0.002, d = 0.87) and uninjured legs (*p* = 0.001, d = 0.78) (Figure 3).

### 2.4. Muscle Thickness of the Biceps Femoris Long Head

**Figure 4:**
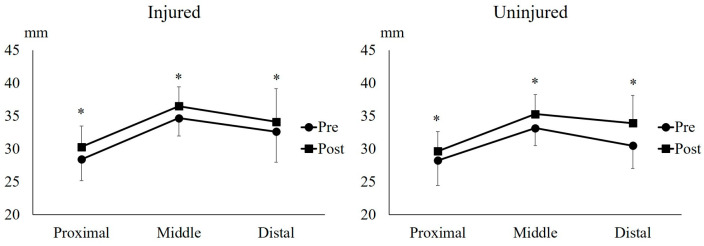
**Proximal, middle, and distal muscle thickness of the biceps femoris long head.** * indicates *p* < 0.05 between Pre and Post.

The Wilcoxon signed-rank test showed that UNHT significantly increased the proximal BFlh muscle thickness (MT) in the injured (*p* = 0.012, r = 0.89) and uninjured legs (*p* = 0.036, r = 0.74) (Figure 4). Furthermore, the Wilcoxon signed-rank test showed that UNHT significantly increased the middle BFlh MT in the injured (*p* = 0.025, r = 0.79) and uninjured legs (*p* = 0.012, r = 0.89) (Figure 4). The paired *t*-test showed that UNHT significantly increased the distal BFlh MT in the injured (*p* = 0.03, d = 0.87) and uninjured legs (*p* = 0.001, d = 0.78) (Figure 4).

### 2.5. Muscle Thickness of the Semitendinosus Muscle

**Figure 5:**
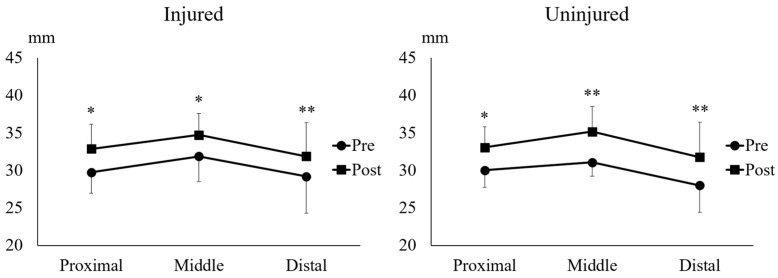
**Proximal, middle, and distal muscle thickness of the semitendinosus muscle.** * indicates *p* < 0.05 between Pre and Post, and ** indicates *p* < 0.01 between Pre and Post.

The Wilcoxon signed-rank test showed that UNHT significantly increased proximal ST thickness in the injured (*p* = 0.012, r = 0.89) and uninjured legs (*p* = 0.012, r = 0.89) (Figure 5). Similarly, the paired *t*-test showed a significant increase in middle ST thickness in the injured (*p* = 0.016, d = 0.92) and uninjured legs (*p* = 0.001, d = 1.51) (Figure 5). The paired *t*-test showed that UNHT significantly increased distal ST thickness in the injured (*p* = 0.002, d = 0.58) and uninjured legs (*p* = 0.000, d = 0.91) (Figure 5).

### 2.6. Muscle Thickness of the Semimembranosus Muscle

**Figure 6:**
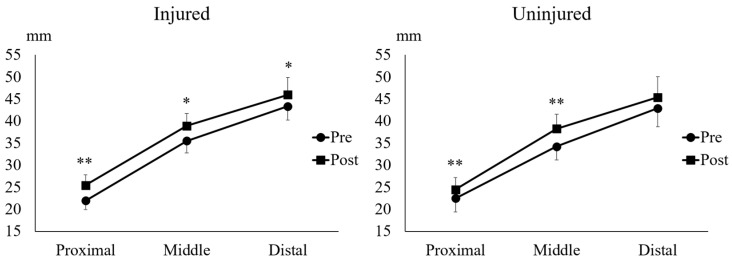
**Proximal, middle, and distal muscle thickness of the semimembranosus muscle.** * indicates *p* < 0.05 between Pre and Post, and ** indicates *p* < 0.01 between Pre and Post.

The paired *t*-test demonstrated that UNHT significantly increased proximal semimembranosus muscle (SM) thickness in the injured (*p* = 0.006, d = 1.58) and uninjured legs (*p* = 0.003, d = 0.76) (Figure 6). Furthermore, it showed that the UNHT significantly increased middle SM thickness in the injured (*p* = 0.011, d = 1.22) and uninjured legs (*p* = 0.004, d = 1.31) (Figure 6). The paired *t*-test showed that UNHT significantly increased distal SM thickness in the injured leg (*p* = 0.018, d = 0.75), but no significant increase was observed in the uninjured leg (*p* = 0.067, d = 0.63) (Figure 6).

## 3. Discussion

These findings are consistent with our hypothesis; however, given the uncontrolled pre–post design and small sample size, the results should be interpreted cautiously. This study investigated the effects of UNHT intervention on BFlh FL in young male athletes with a history of HSI. UNHT led to a significant increase in the middle BFlh FL in the injured and uninjured legs. However, only the injured leg showed a significant increase in distal BFlh FL. These results support our hypothesis.

Athletes with a history of HSI often exhibit a shorter BFlh FL in the injured leg than in the uninjured one [5,6]. Research has indicated that a shorter BFlh FL correlates with a higher incidence of HSI [7], suggesting that the high recurrence rate of HSI is highly likely due to this fascicle shortening in the injured leg. This study demonstrated that the UNHT intervention significantly lengthened the middle (Figure 1) and distal BFlh FL (Figure 2) in the injured leg. By contrast, although middle BFlh FL lengthening (Figure 1) was significant in the uninjured leg, no notable lengthening was observed in the distal BFlh FL (Figure 2). Furthermore, the adaptation in the middle BFlh FL was greater in the injured leg than in the uninjured leg (Figure 1). This quantified difference-in-change (11.4 mm, 95% CI: 1.4 to 21.3 mm) supports the interpretation that the injured leg exhibited greater fascicle length adaptation than the uninjured leg. This discrepancy in fascicle adaptation may be attributed to changes in extracellular matrix components within the injured muscle [13]. A study reported that α^7^β^1^-integrin, a protein associated with the extracellular matrix, increases after high-intensity eccentric exercise that induces muscle damage [14]. This study also reported that elevated levels of α^7^β^1^-integrin promote the phosphorylation of threonine 389 in p70S6K, a key factor in activating protein synthesis for skeletal muscle growth [15]. As an adaptation to HSI, extracellular matrix components, such as α^7^β^1^-integrin, might have been modified in the injured leg, potentially leading to greater BFlh FL adaptation in response to the UNHT intervention than in the uninjured leg. Since unilateral isokinetic eccentric training involving shallow knee flexion prevents HSI recurrence [16], UNHT conducted in this study also has the potential to prevent HSI recurrence. However, a longer intervention study is required to determine whether UNHT can prevent HSI recurrence. Although alterations in extracellular matrix components and α^7^β^1^-integrin signaling provide a plausible explanation for the greater fascicle length adaptation observed in the injured leg, this interpretation is speculative because the present study did not include biomarker or tissue-level assessments. Therefore, this mechanistic explanation should be considered hypothesis-generating. Alternative explanations are also possible, including baseline architectural differences between legs, subtle differences in training execution, measurement or analysis bias, and regression to the mean.

Meta-analyses have revealed a significant predictive effect of absolute eccentric knee flexor strength at 60°/s for detecting the risk of future HSI [17]. Therefore, the results from the eccentric knee flexion strength test in this study seem to reflect future HSI risk. The results of this study demonstrated a significant improvement in eccentric knee flexor strength in the injured and uninjured legs (Figure 3). In a 16-week preseason training program, standard bilateral NHE was included for athletes with and without a history of HSI [18]. Consequently, eccentric knee flexor strength improved by 26% in athletes with no history of HSI, compared with only a 7% increase in those with a history of HSI. In the present study, eccentric knee flexor strength improved by 14% in the injured leg and 10% in the uninjured leg after 6 weeks of UNHT. Given that previous research has indicated that unilateral training may yield greater improvements in muscle strength than bilateral training [19], UNHT may produce a more substantial effect on improving eccentric knee flexor strength than standard NHE. Further investigation into the comparative training effects of UNHT and standard NHE is warranted in future studies. Although these comparisons provide useful context, the protocols differed substantially across studies, including intervention duration, weekly training volume, unilateral versus bilateral execution, and the use of a sloped platform in the present study. Testing procedures also varied, such as the use of isokinetic dynamometry at 60°/s in our study versus alternative devices or velocities in prior work. To improve interpretability, we now report absolute changes in eccentric knee flexor strength (Nm), and these values may be further interpreted relative to body mass in future research.

Since the BFlh accounts for 60–100% of HSIs [1,2], adapting this muscle through training is important to reduce the risk of HSI recurrence. In addition to a shortening of the BFlh FL, BFlh atrophy is commonly observed in athletes with a history of HSI [20]. A study reported that BFlh injuries occur equally in the proximal, middle, and distal regions [21], with prominent muscle atrophy observed at the injury site [20]. Findings from this study demonstrated that UNHT significantly increased MT in the proximal, middle, and distal regions of the BFlh in both the injured and uninjured legs (Figure 4), indicating that UNHT caused muscle hypertrophy throughout the BFlh. BFlh MT has potential as a future risk factor for HSI, but conclusive evidence on its influence is lacking. Further research exploring the relationship between BFlh MT and HSI risk is warranted. Although increases in muscle thickness may reflect hypertrophic adaptations, ultrasound-derived MT is an indirect proxy that does not capture fascicle length, pennation angle, or functional muscle behavior. Therefore, conclusions regarding the potential role of MT in preventing HSI recurrence should be interpreted cautiously, and future studies incorporating more comprehensive architectural and functional assessments are warranted.

ST injury is the second most common hamstring strain after BFlh injury and similarly occurs in the proximal, middle, and distal regions [21]. However, to our best knowledge, no studies to date have reported ST atrophy at the injury site. Thus, whether ST hypertrophy in the proximal, middle, and distal regions of the injured leg can prevent HSI recurrence is unknown. Results from the current study demonstrated significant increases in ST thickness in the proximal, middle, and distal sections in the injured and uninjured legs following UNHT (Figure 5). Although ST thickness could become a potential future risk factor for HSI, current evidence remains inconclusive. Further research examining the relationship between ST MT and HSI risk is recommended.

Most stretch-type HSIs are SM injuries [22]. Severe proximal SM injury can result in SM atrophy [23], making it important to prevent HSI recurrence through promoting SM hypertrophy. The results of the present study demonstrated that UNHT significantly increased SM thickness in the injured and uninjured legs, especially in the proximal and middle regions (Figure 6). Notably, only the injured leg showed a significant increase in SM thickness in the distal region (Figure 6). Although a significant trend was observed in the distal SM thickness of the uninjured leg, extending the intervention period may lead to adaptations in the uninjured leg that mirror those in the injured leg. Given that the relationship between SM muscle size and HSI risk is unknown, further research exploring the relationship between SM thickness and HSI risk may be necessary.

There are several limitations to this study. First, the sample size was small (n = 8), which limits statistical power and reduces the generalizability of the findings. Although the sample size was justified based on prior work, replication in larger and more heterogeneous cohorts—across different ages, sexes, and performance levels—is needed. Second, the history of HSI, including diagnosis, severity, and rehabilitation details, was based on self-reported information, introducing the possibility of misclassification. Third, the study lacked a control or comparator group, which restricts causal inference regarding the effects of the UNHT intervention. Fourth, limb-level data are not statistically independent; although within-subject analyses were applied, this non-independence remains an inherent limitation of the study design. Fifth, muscle architecture was assessed in a relaxed prone posture, which may not fully reflect functional behavior during high-speed running or sprinting. Sixth, given the large number of outcomes examined, the potential for Type I error must be acknowledged. While the middle BFlh fascicle length of the injured leg was defined a priori as the primary outcome, all other variables were secondary or exploratory. Because no correction for multiple comparisons was applied to these exploratory analyses, some significant findings may represent false positives and should be interpreted with caution. Finally, ultrasound images were analyzed by a single investigator who was not blinded to time point or limb condition, which may have introduced measurement bias.

## 4. Materials and Methods

### 4.1. Experimental Approach to the Problem

This longitudinal study investigated the effects of UNHT on the BFlh FL in young males with a history of hamstring injury. Pre-training measurements included assessments of the hamstring muscle architecture and eccentric knee flexion strength for the injured and uninjured legs. Concerning the hamstring architecture, measurements were taken for the BFlh FL and MT of the hamstring muscles. Furthermore, the BFlh FL was measured at the middle and distal regions, whereas MT was measured at the proximal, middle, and distal regions. Post-training measurements mirrored these pre-measurement assessments. Based on prior literature demonstrating its relevance to HSI risk and remodeling, the middle BFlh fascicle length of the injured leg was designated as the primary endpoint of the study. All other architectural and strength measures were considered secondary or exploratory outcomes.

### 4.2. Participants

G*Power software (version 3.1; Heinrich Heine Universität, Düsseldorf, Germany) was used to estimate the required sample size. The sample size was calculated a priori based on parameters (effect size = 1.25, alpha = 0.05, power = 0.8) derived from previous studies [5,24]. Consequently, a minimum of eight participants was required. The inclusion criteria were as follows: (1) age between 18 and 35 years, (2) a history of HSI within the past 2 years, and (3) HSI affecting only one leg. HSI was defined as sudden pain in the posterior thigh that occurred during athletic activities, such as soccer, basketball, taekwondo, or sepaktakraw (kick volleyball). Detailed information regarding injury diagnosis, location, severity, and rehabilitation after HSI was not available due to self-reporting. Although participants reported experiencing “sudden posterior thigh pain during sport,” this definition is broad and may encompass non-hamstring pathologies. Although some athletes were evaluated by medical staff at the time of injury, formal diagnostic documentation was not consistently available. Therefore, the possibility of misclassification, including non-hamstring sources of posterior thigh pain or variation in injury severity, must be acknowledged. The classification of HSI as sprint- or stretch-related followed the categorization framework used in a previous study [2]. Exclusion criteria included any lower or upper extremity injuries that could limit the participant’s ability to perform UNHT. The experimental protocol received approval from the Institutional Review Board of Mahidol University (MU-CIRB 2023/226.1407). All procedures were performed in accordance with the Declaration of Helsinki. All participants were informed of the purpose and procedure of the study, and informed consent was obtained from them.

### 4.3. Procedures

For pre-training and post-training measurements, the middle and distal regions of the BFlh FL and the proximal, middle, and distal regions of MT of the hamstring muscles were initially measured using ultrasonography. Ultrasonograms were captured twice. Participants performed a static hamstring stretch (a standing hamstring stretch on one leg) with a 20-s hold on each leg. After stretching, participants completed a specific warm-up for eccentric knee flexion strength testing, consisting of two sets of three repetitions at submaximal subjective effort. In the first set, the force output was held at 20% subjective effort, while in the second set, subjective effort levels were gradually increased to 20%, 50%, and 80%. After this warm-up, participants performed an eccentric knee flexion strength test, consisting of two sets of three consecutive maximal-effort repetitions, with a 1-min rest between sets. During the eccentric knee flexor strength test, measurements were taken in a randomized order for the injured and uninjured legs. Post-training measurements were conducted at least 2 days after the final training session and within 6 days (3.3 ± 1.5 days).

Participants performed UNHT twice a week for 6 weeks, for a total of 12 sessions. After the same hamstring stretch as performed in the pre-training and post-training measurements, participants completed three repetitions of UNHT as a specific warm-up, with subjective effort levels gradually increased from 50% to 80% and then to 100%. Then, they performed three sets of eight repetitions, with a rest of 5–10 s between repetitions. Training was conducted for both the injured and uninjured legs, with a 1-min rest between sets and a 2-min rest between legs. The order of limb training was randomized across sessions to avoid systematic bias related to fatigue or learning effects. Each training session was separated by at least 1 day.

Sloped-platform angles were recorded at every session, allowing us to document progression across the 6-week intervention. Angle adjustments occurred most frequently during the first 2–3 weeks, after which angles tended to stabilize. Progression patterns were similar between limbs, and no systematic differences in angle adjustments were observed between the injured and uninjured legs. Although BPA was used to guide progression, BPA values were not recorded across sessions; therefore, only sloped-platform angles are summarized in Table 1.

### 4.4. Hamstring Architecture

To identify the measurement locations of the proximal, middle, and distal hamstring muscles, thigh length was measured in the injured and uninjured legs. Thigh length was defined as the distance from the greater trochanter (0% of thigh length) to the popliteal crease (100% of thigh length). Measurement locations were marked along the thigh axis at 35%, 50%, and 65% of the total thigh length, corresponding to the proximal, middle, and distal regions, respectively [25]. Participants assumed a prone position on a trainer bed, with hip and knee joints fully extended, and rested for 10 min before the assessment. Images were then captured using a B-mode ultrasound system (Philips Lumify, Bothell, WA, USA) with a L12–4 linear probe (frequency, 4–12 MHz; width, 3.4 cm; depth, 8.0 cm). After applying a transmission gel between the skin and scanner, the probe was placed at the measurement locations with minimal pressure. The same examiner performed the ultrasound measurements. The ultrasound probe was completely removed from the skin after the initial measurement, and a second measurement was performed. All ultrasonograms were analyzed using Tracker software (version 6.2.0) [26].

**Figure 7:**
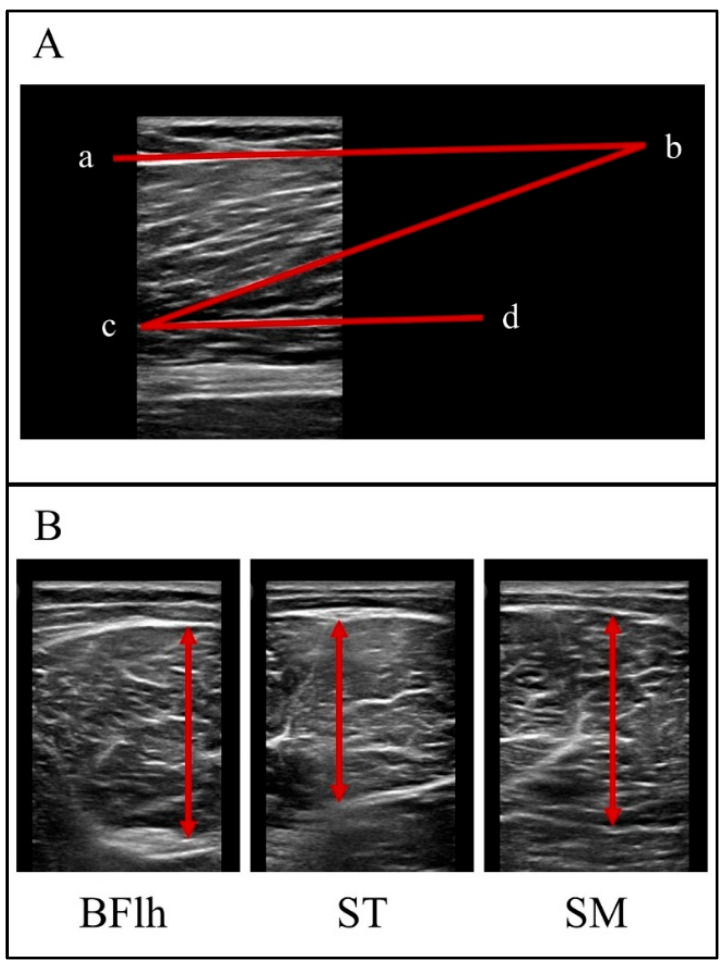
**Analysis method for the ultrasonograms.** (**A**) The BFlh FL was measured at 50% of the thigh length. Lines (ab) and (cd) indicate the positions of the superficial and intermediate aponeuroses, respectively. The line (bc) indicates the FL. (**B**) Muscle thickness of the BFlh, ST, and SM was measured at 50% of the thigh length. Arrows indicate the thickness of each muscle.

The BFlh FL was measured using a probe positioned along the longitudinal axis of the muscle. Scanning locations were determined at 50% and 65% of the thigh length. In many images, the transducer could not capture the entire fascicle length; therefore, the superficial and intermediate aponeurosis lines were extrapolated beyond the image frame. Fascicle length was defined as the linear distance between the points where the fascicle intersected the extrapolated lines of the superficial and intermediate aponeuroses (Figure 7A) [26]. MT was measured with the probe positioned on the crosswise axis, with scans taken at 35%, 50%, and 65% of thigh length. Target muscles included the BFlh, ST, and SM. MT was defined as the maximum distance between the superficial and deep fasciae (Figure 7B) [27].

Fascicle length was calculated by manually identifying a clearly visible fascicle located near the center of the image. When either the superficial or deep aponeurosis extended beyond the ultrasound image frame, the visible portion of each aponeurosis was fitted with a straight line and linearly extrapolated outside the field of view. Fascicle length was then determined as the linear distance between the intersection points of the selected fascicle and the extrapolated aponeuroses. The same anatomical region was imaged pre- and post-intervention, and probe placement was reproduced using the same anatomical landmarks and orientation. The region of interest and analysis procedures were identical across time points.

### 4.5. Eccentric Knee Flexion Strength

Eccentric knee flexion strength was measured using an isokinetic dynamometer (Biodex System 4; Biodex Medical Systems, Shirley, NY, USA). The examiner informed the participants about the characteristics of the dynamometer. During testing, participants were seated with the hip flexed at an 85° angle (where 0° represents a fully extended hip). The rotational axis of the dynamometer was aligned with the lateral epicondyle of the femur, and the shin pad of the lever arm was placed above the medial malleolus. To ensure stability, straps were tightened around the participant’s chest, pelvis, and thighs. The range of motion for testing was set between 20° and 90° of knee flexion (where 0° represents a fully extended knee), with an angular velocity of 60°/s as an indicator of HSI risk [17]. Gravity correction was applied at 30° of knee flexion. Participants were instructed to hold the dynamometer bars with both hands during testing and received consistent verbal encouragement throughout the test.

### 4.6. Nordic Hamstring Training

**Figure 8:**
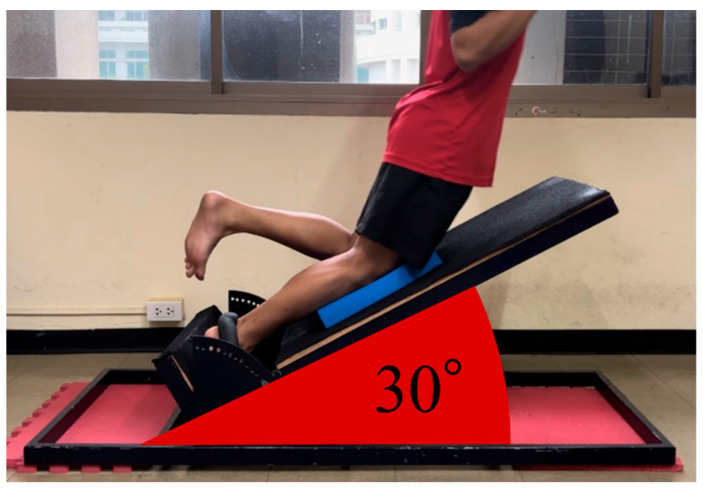
**Depiction of unilateral Nordic hamstring training.** The sloped angle is set at 30°, and the participant is unilaterally performing with the right leg.

A custom-designed sloped platform with variable angles was used (Figure 8). Participants began in a kneeling position with their elbows fully flexed and their hands open in front of them. A non-elastic band with soft rubber cushions secured each participant’s ankles to the platform. Participants were instructed to keep their bodies straight from the knees to the head and to avoid trunk rotation during the exercise. They were also instructed to keep the knee of the non-tested leg in contact with the platform while allowing the foot to move away. The examiner then directed participants to lean forward “as slowly as possible.” The initial slope angle of the platform was set to 40°, which facilitated the recruitment of the BFlh [12]. If the BPA was within 30° by the third repetition of a specific warm-up, the platform angle was reduced by 10°. The Nordic Angle application was used to measure the BPA [28].

### 4.7. Data Analysis

All ultrasound measurements of the hamstring muscles were averaged across two data points. The intraclass correlation coefficients(1,1) and coefficients of variation were as follows: 0.89 and 3.4% for the middle BFlh FL, 0.84 and 3.9% for the distal BFlh FL, 0.98 and 1.3% for the proximal BFlh MT, 0.97 and 1.3% for the middle BFlh MT, 0.99 and 1.2% for the distal BFlh MT, 0.97 and 1.5% for the proximal ST thickness, 0.97 and 1.3% for the middle ST thickness, 0.97 and 2.0% for the distal ST thickness, 0.94 and 2.4% for the proximal SM thickness, 0.95 and 1.7% for the middle SM thickness, and 0.96 and 1.3% for the distal SM thickness, respectively.

Typical error (TE) was calculated as the standard deviation of the difference between repeated measurements divided by √2. Minimal detectable change (MDC) was derived from TE using the formula MDC = TE × 1.96 × √2, representing the smallest change that exceeds the measurement error with 95% confidence. Across all ultrasound variables, TE values ranged from 0.17 to 5.07 mm, and MDC values ranged from 0.46 to 14.06 mm. Pre–post changes in each variable were compared against the corresponding TE and MDC values to evaluate whether observed differences exceeded the measurement error and therefore reflected practically meaningful changes.

The eccentric knee flexion strength test was performed with two sets of three consecutive repetitions. The peak torque value for each set was determined as the highest torque recorded among the three repetitions, and these two peak torque values were then averaged. The intraclass correlation coefficient(1,1) and coefficient of variation for eccentric knee flexion strength were 0.94 and 2.8%, respectively.

### 4.8. Statistical Analysis

Data are expressed as mean ± standard deviation. The Shapiro–Wilk test was used to assess normality. The Shapiro–Wilk test indicated that several variables did not meet the assumption of normality. Specifically, distal BFlh FL, proximal BFlh MT, middle BFlh MT, and proximal ST MT were non-normally distributed. Accordingly, these variables were analyzed using the Wilcoxon signed-rank test. To evaluate the main or interaction effects of leg (injured vs. uninjured; within-subject factor) and time (pre versus [vs.] post; within-subject factor) on the BFlh FL, MT of the hamstring muscles, and eccentric knee flexion strength, a two-way repeated-measures ANOVA (leg × time) was performed. This analysis was restricted to parametric data. When an interaction effect was observed, post hoc comparisons were conducted using the Bonferroni test. Partial η^2^ values were classified according to effect size criteria as follows: trivial (<0.01), small (0.01–0.06), medium (0.06–0.14), and large (>0.14). Within-group changes from pre-training to post-intervention (pre vs. post; within-subject factor) were analyzed using a paired *t*-test for parametric data and the Wilcoxon signed-rank test for non-parametric data. Effect sizes were calculated using Cohen’s d for parametric tests and interpreted as trivial (<0.2), small (0.2–0.6), medium (0.6–1.2), large (1.2–2.0), and very large (>2.0). For Wilcoxon signed-rank tests, nonparametric effect sizes were calculated using r (r = Z/√N) and interpreted as trivial (<0.10), small (0.10–0.29), medium (0.30–0.49), and large (≥0.50). All statistical analyses were performed using SPSS (version 29; IBM Corp., Armonk, NY, USA). Statistical significance was set at *p* < 0.05. To address the issue of multiple comparisons, we defined the middle BFlh fascicle length of the injured leg as the primary outcome a priori, based on its clinical relevance and previous evidence indicating its sensitivity to architectural remodeling after HSI. All other outcomes (additional BFlh sites, muscle thickness measures, and eccentric strength) were treated as secondary or exploratory outcomes. No statistical correction for multiple comparisons was applied to these secondary/exploratory analyses; therefore, the results should be interpreted with caution due to the increased risk of Type I error.

## 5. Conclusions

This study demonstrated that unilateral Nordic hamstring training with a sloped platform increased BFlh fascicle length and eccentric knee flexion strength in athletes with a history of HSI. Because shorter BFlh fascicle length and reduced eccentric strength are associated with a higher risk of HSI, these adaptations may have implications for injury risk reduction. However, this study did not evaluate recurrence rates, and controlled prospective studies are required to determine whether UNHT can reduce HSI recurrence.

## Figures and Tables

**Table 1:** Sloped-platform angle (°) used during each training session for the injured and uninjured limbs. Values are presented as mean ± standard deviation (SD). These data summarize the progression of sloped-platform angles across the 12 unilateral Nordic hamstring training sessions and demonstrate that progression patterns were similar between limbs.

Week	Injured Leg (°)	Uninjured Leg (°)
Week 1	29.4 ± 2.4	29.4 ± 2.4
Week 2	25.0 ± 5.0	24.4 ± 5.0
Week 3	21.3 ± 3.3	21.3 ± 3.4
Week 4	20.0 ± 0.0	20.0 ± 0.0
Week 5	18.1 ± 3.9	18.8 ± 3.3
Week 6	16.3 ± 4.8	16.9 ± 4.6

## Data Availability

The datasets generated and/or analyzed during the current study are not publicly available but are available from the corresponding author, who organized the study.

## Abbreviations

The following abbreviations are used in this manuscript:

HSIs	Hamstring strain injuries
BFlh	Biceps femoris long head
BFlh FL	Fascicle length of the biceps femoris long head
NHE	Nordic hamstring exercise
BPA	Break-point angle
ST	Semitendinosus muscle
UNHT	Unilateral Nordic hamstring training
ANOVA	Analysis of variance
MT	Muscle thickness
vs.	Versus
SM	Semimembranosus muscle
